# FP-ZOO: Fast Patch-Based Zeroth Order Optimization for Black-Box Adversarial Attacks on Vision Models

**DOI:** 10.3390/s25227093

**Published:** 2025-11-20

**Authors:** Junho Seo, Seungho Jeon

**Affiliations:** 1 Telecommunications Technology Association, Bundang-ro 47, Bundang-gu, Seongnam-si 13591, Gyeonggi-do, Republic of Korea; jhseo@tta.or.kr; 2 Department of Smart Security, Gachon University, Seongnam-daero, Sujeong-gu, Seongnam-si 1332, Gyeonggi-do, Republic of Korea

**Keywords:** adversarial attack, evasion attack, black-box attack, zeroth order optimization, vision model

## Abstract

Deep neural networks have outperformed conventional methods in various fields such as image recognition, natural language processing, and speech recognition. In particular, vision models are widely applied to real-world domains including medical image analysis, autonomous driving, smart factories, and security surveillance. However, these models are vulnerable to adversarial attacks, which pose serious threats to safety and reliability. Among different attack types, this study focuses on evasion attacks that perturb the inputs of deployed models, with an emphasis on black-box settings. The zeroth order optimization (ZOO) attack can approximate gradients and execute attacks without access to internal model information, but it becomes inefficient and exhibits low success rates on high-resolution images due to its dependence on image resizing and its high memory complexity. To address these limitations, this study proposes a patch-based fast zeroth order optimization attack, FP-ZOO. FP-ZOO partitions images into patches and generates perturbations effectively by employing probability-based sampling and an ϵ-greedy scheduling strategy. We conducted a large-scale evaluation of the FP-ZOO attack on the CIFAR-10, CIFAR-100, and ImageNet datasets. In this evaluation, we adopted attack success rate, $L_{2}$ distance, and adversarial example generation time as performance metrics. The evaluation results showed that the FP-ZOO attack not only achieved an attack success rate of 97–100% against ImageNet in untargeted attacks, but also demonstrated performance up to 10 s faster compared to the ZOO attack. However, in targeted attacks, it showed relatively lower performance compared to baseline attacks, leaving it as a future research topic.

## 1. Introduction

Recent advances in deep neural networks have demonstrated superior performance compared to traditional machine learning techniques across various domains, including image recognition, natural language processing, and speech recognition. In the field of computer vision in particular, remarkable progress has been achieved in tasks such as object detection, image classification, and scene understanding, which has extended to real-world applications such as medical image analysis, autonomous driving, smart factories, and security surveillance [1]. However, these vision models have an inherent vulnerability to adversarial attacks [2]. In safety-critical domains, such attacks can result in physical accidents or security breaches, highlighting the severity of this issue [3].

Adversarial attacks refer to a collection of techniques designed to compromise deep neural networks and are generally categorized into four types: evasion, poisoning, extraction, and inference attacks. Among these, an evasion attack perturbs the input after the model has been deployed to induce incorrect outputs. Depending on the amount of information the attacker possesses about the model, evasion attacks are classified as white-box or black-box attacks [4]. Furthermore, evasion attacks can be divided into targeted and untargeted attacks based on their objectives. This study focuses on black-box evasion attacks against vision models, and unless otherwise specified, the terms adversarial attack and evasion attack are used interchangeably.

Many black-box adversarial attacks have been proposed to date. The zeroth order optimization (ZOO) attack [5] is a technique that approximates gradients using only model inputs and outputs in black-box settings where internal structure is unknown. It does not require training a substitute model, which saves time and resources and avoids issues related to transferability loss [6]. Moreover, through dimensionality reduction, hierarchical attacks, and importance sampling, ZOO enables efficient optimization for high-dimensional inputs. However, in this study we point out that ZOO becomes inefficient and exhibits substantially reduced success rates for large-size images.

More specifically, we analyze two reasons why the ZOO attack is inefficient for large images. ❶ Perceptibility: the ZOO attack employs image resizing to perform attacks on large images both effectively and efficiently. Consequently, the success rate of ZOO on large images depends more on the resizing procedure than on the algorithmic design of ZOO itself. However, because adversarial attacks fundamentally aim to generate adversarial examples that are visually indistinguishable from the original images, ZOO’s reliance on resizing contradicts this core objective. ❷ Memory complexity: when attacking large images without resizing, the space complexity of coordinate descent optimization increases substantially, causing the attack to become very slow. In other words, ZOO consumes excessive memory in practice.

To overcome these limitations of the ZOO attack, this paper proposes FP-ZOO, a patch-based fast zeroth order optimization attack against vision models. Unlike ZOO, FP-ZOO partitions the image into multiple patches instead of downscaling it. Then, perturbations are computed and injected into the image via stochastic coordinate descent applied at the patch level. By doing so, FP-ZOO effectively reduces the search space and attacks the model efficiently without resizing the image. In addition, we propose a patch-sampling method that computes a probability distribution over patches to prioritize those likely to be effective for the attack. We also introduce an ϵ-greedy scheduling strategy to ensure that no single patch is repeatedly selected and to allow all patches some chance of selection. In large-scale experiments across multiple datasets, FP-ZOO achieved rapid success in untargeted attacks due to its computational efficiency, while it exhibited limitations in targeted attacks because of coarse gradient estimation. This research can be utilized to effectively reveal the vulnerabilities of vision models against perturbations, and the adversarial examples generated by the FP-ZOO attack can be used to enhance the robustness and reliability of the models.

The contributions of this paper are threefold.

- We propose a patch-based zeroth order optimization attack that enables effective black-box evasion attacks on large-size images.
- We introduce probability-based patch sampling to inject perturbations efficiently and an ϵ-greedy scheduling strategy to control sampling randomness.
- We conduct large-scale evaluations of the proposed attack across multiple datasets containing images of various sizes; experimental results show that the proposed method outperforms existing zeroth order optimization-based attacks in untargeted attacks.

The remainder of this paper is organized as follows. Section 2 reviews prior research on adversarial attacks, distinguishing between white-box and black-box settings; Section 3 describes in detail the attack techniques that form the basis of the FP-ZOO attack; Section 4 proposes the patch-based fast zeroth order optimization algorithm; Section 5 provides a thorough evaluation of FP-ZOO across multiple datasets, vision models, and baseline attack techniques; Section 6 discusses several limitations of FP-ZOO revealed by the evaluation and potential strategies to address them; and Section 7 concludes the paper with a summary of the proposed method and directions for future research.

## 2. Related Work

Adversarial attacks have been actively studied since deep neural networks were deployed across various application domains. In particular, adversarial attacks can be classified as black-box or white-box depending on the information available to the adversary. In this section, we list representative attacks in each category and describe the characteristics of each attack.

### 2.1. White-Box Adversarial Attacks

White-box attacks assume that the adversary has full access to the target model. Leveraging this advantage, many white-box attacks compute gradients of the attack objective directly and generate adversarial examples based on those gradients. The Fast Gradient Sign Method (FGSM) [7] is a single-step attack that exploits the linear behavior of neural networks in high-dimensional spaces by applying a small perturbation in the direction of the loss gradient. Because it requires only one backpropagation pass to generate an example, FGSM is computationally efficient, applicable to various models and datasets, and can be used to improve model generalization when adversarial examples are included during training as a form of regularization. However, FGSM’s attack strength is limited compared to iterative or optimization-based methods, and the choice of perturbation magnitude creates a trade-off between attack success rate and detectability that can limit practical applicability. The Jacobian-based Saliency Map Attack (JSMA) [8] evaluates the influence of individual features on the target class probability using the Jacobian matrix of outputs with respect to inputs, and iteratively perturbs a small set of highly influential features to steer the classifier toward the target class. JSMA can achieve high success rates with few pixel changes, offering computational efficiency and interpretability, and has proven effective on low-dimensional datasets. Its scalability is limited, however, since computational cost and search space grow rapidly for high-dimensional images or large datasets, restricting its use on real-world high-resolution inputs. DeepFool [9] is an iterative attack that locally linearizes the classifier decision boundary to approximate the minimal perturbation required to change the input’s classification. DeepFool produces much smaller perturbations than single-step methods like FGSM, resulting in more precise and realistic adversarial examples, but its iterative linearization is computationally expensive and can be inaccurate in regions with strong nonlinearity. Moreover, its design centered on the $L_{2}$ norm limits direct optimization under other norms. The Carlini and Wagner (C&W) attack [10] is a white-box optimization method that formulates and solves an optimization problem to minimize distance to the original input under various constraints, generating highly refined adversarial examples. Its optimization-based design achieves high precision and good evasion capability against many defenses proposed at the time, establishing C&W as a strong attack benchmark. However, it requires careful tuning of the objective and balancing parameters and incurs high computational cost due to iterative optimization. Auto-PGD [11] extends projected gradient descent (PGD) [12] by incorporating adaptive step sizes and automatic stopping criteria, enabling strong attacks without manual parameter tuning. By effectively exploring diverse initializations and parameter combinations, Auto-PGD improves the stability and reproducibility of evaluations, though it remains computationally heavier than single-step methods because it is iterative.

### 2.2. Black-Box Adversarial Attacks

Black-box attacks assume that the adversary does not have access to the target model and that only inputs and model outputs are available. Despite this limitation, black-box attacks represent a practical approach for attacking commercial models or evaluating robustness, and thus they have been extensively studied. The ZOO attack [5] performs optimization-based attacks by approximating gradients using only input–output queries when internal model information is unavailable. By combining techniques such as stochastic per-coordinate search, importance sampling, and dimensionality reduction, ZOO is designed to achieve strong attack performance comparable to the C&W attack even on high-dimensional inputs. However, because gradient approximation and optimization in ZOO rely on queries, it may require hundreds of thousands of model calls, creating a practical constraint due to high query cost in real environments. The Boundary attack [13] operates under the restricted setting where only class labels are observable. It begins from an adversarial example with a large perturbation and uses random walks and rejection sampling to progressively reduce perturbation while preserving adversarial properties. Because it has access only to the final label and no additional information, the Boundary attack similarly incurs very high computational and query costs, often requiring hundreds of thousands of queries. The Simple Black-box Attack (SimBA) [14] is a straightforward search-based method that, without gradient information, iteratively updates the input along randomly chosen directions from a predefined set using only confidence scores returned by the model. SimBA’s simple design yields high query efficiency, achieving high success rates on ImageNet and commercial-model settings with far fewer queries than many prior black-box methods, making it a practical baseline. Its effectiveness, however, depends on the availability of continuous confidence scores and is limited in hard-label settings that return only final labels. The HopSkipJump Attack (HSJA) [15] is designed for decision-based environments. Starting from an initial adversarial example, it uses binary search to find a point near the decision boundary and then leverages gradient estimates obtained via queries to iteratively reduce the perturbation. Compared to earlier boundary-based approaches that relied on random exploration, HSJA improves query efficiency by orders of magnitude and can reach minimal perturbations in hard-label settings with relatively fewer queries. Nevertheless, it still requires a large number of queries, and its computational cost grows substantially for very high-dimensional, high-resolution inputs. The Square attack [ref-square-attack] adopts a simple random-search strategy that repeatedly modifies square-shaped patch regions rather than performing complex gradient estimation or optimization. This approach is both simple and efficient, achieving higher success rates per query than many previous black-box attacks across diverse datasets and maintaining robust performance even in defended environments. Its reliance on random search, however, can make performance unstable if initial queries are insufficient, and it is limited in decision-only environments that provide only output labels.

## 3. Preliminary

This section provides the background necessary to describe the FP-ZOO attack in detail. FP-ZOO is fundamentally derived from the ZOO attack. Moreover, the ZOO attack is a black-box adaptation of the white-box C&W attack and shares many design elements, including the objective. Therefore, we treat the C&W attack and the ZOO attack as the preliminaries for FP-ZOO.

### 3.1. Carlini and Wagner Attack

Unlike earlier adversarial attacks such as FGSM or DeepFool, the C&W attack aims to generate adversarial examples that fool the model with minimal distortion. To achieve this, it formulates adversarial example generation as an optimization problem rather than simply computing the gradient of the objective with respect to the input x. Concretely, the C&W attack is designed to find a solution that satisfies two conditions: ❶ the adversarial example should be similar to the original input, and ❷ it should cause the model to fail its prediction, which is the fundamental goal of an adversarial attack. For simplicity, this paper assumes a classification model for image data as the target model. To realize these objectives, the C&W attack defines an objective as in Equation (1).

(1) $$\begin{array}{cc} {\hat{x}}^{*} & =\arg_{\hat{x}}\min||\hat{x}-x{\left|\right|}_{2}^{2}+c\cdot f\left(\hat{x},t\right) \end{array}$$

where $\hat{x}$ and *t* denote the adversarial example and the target class, respectively. The scalar *c* is a weight that balances similarity and attack success. The first and second terms on the right-hand side of this objective correspond to goals ❶ and ❷, respectively. More specifically, the first term enforces that the adversarial example remains close to the input x by using the $L_{2}$ distance. This term encourages small, visually imperceptible perturbations, thereby reducing perceptibility; in other words, it is effective at producing adversarial examples that are visually similar to the original image x. N. Carlini and D. Wagner [10] also adopted $L_{0}$ and $L_{\infty }$ distances as similarity metrics in addition to the $L_{2}$ distance. The $L_{0}$ distance counts the number of coordinates (pixels) changed between the original image x and the adversarial example $\hat{x}$, focusing on how many pixels are altered by the attack rather than on Euclidean distance. The $L_{\infty }$ distance is computed as the maximum change among all pixels, effectively constraining the maximum allowable change per pixel. While each distance has its own characteristics, this paper proceeds with the $L_{2}$ distance for the remainder of the discussion. Although the $L_{2}$ distance forces x and $\hat{x}$ to be close in the input space, it does not by itself ensure attack success; the second term on the right-hand side of Equation (1) serves that purpose.

(2) $$\begin{array}{cc} f(\hat{x},t) & =\max\{\max_{i\neq t}Z_{i}\left(\hat{x}\right)-Z_{t}\left(\hat{x}\right),-\kappa \} \end{array}$$

Equation (2) defines $f(\hat{x},t)$ used in targeted attacks (the objective for untargeted attacks can be readily derived from the targeted-attack objective. However, for clarity of exposition, we describe the C&W attack based on the targeted attack). Where $Z_{t}\left(x\right)$ denotes the logit value for class *t* in the model’s output on input x. κ is a hyperparameter that controls the margin and thus the attack strength. The term $\max_{i\neq t}Z_{i}\left(\hat{x}\right)-Z_{t}\left(\hat{x}\right)$ computes the difference between the largest logit among classes other than the target and the target-class logit for the adversarial example $\hat{x}$. In other words, when $Z_{t}\left(\hat{x}\right)$ exceeds $\max_{i\neq t}Z_{i}\left(\hat{x}\right)$, the targeted attack is considered successful. The objective enforces that this logit difference becomes less than −κ; by requiring the difference to be below −κ, the attack strength is controlled. For example, if κ is 0, the attack may stop once the model’s largest non-target logit is no greater than the target logit for a given input x.

As noted earlier, the C&W attack reduces adversarial example generation to an optimization problem, unlike conventional attack methods. More specifically, rather than searching directly for the perturbation to be applied to the original image, the C&W attack constructs the adversarial example directly by employing a variable-substitution trick. This trick is introduced because the input x is constrained ($x\in {\left[0,1\right]}^{p}$), which would otherwise require solving a constrained optimization problem. Introducing a new variable *w* allows the objective in Equation (1) to be transformed into an unconstrained optimization problem.

(3) $$\begin{array}{cc} \hat{x} & =\frac{1}{2}\left(\tanh\left(w^{*}\right)+1\right)\\ s.t.\;w^{*} & =\arg_{w}\min||\frac{1}{2}\left(\tanh\left(w\right)+1\right)-x{\left|\right|}_{2}^{2}+c\cdot f\left(\frac{1}{2}\left(\tanh\left(w\right)+1\right),t\right) \end{array}$$

Equation (3) presents the C&W objective with the variable-substitution trick incorporated. The equation is quite straightforward. The tanh function takes values between −1 and 1, so tanh(w)+1 lies in the range from 0 to 2; multiplying this by $\frac{1}{2}$ maps the result to between 0 and 1, thereby ensuring that x always remains within the valid input range. Consequently, we can ignore the box constraint on x and optimize the objective with respect to *w* to obtain the optimal adversarial example $\hat{x}$. Gradient descent can be used to perform the optimization over *w*.

### 3.2. Zeroth Order Optimization Attack

Building on the C&W attack formulation above, we now discuss the ZOO attack. ZOO is fundamentally designed to perform the C&W attack in a black-box setting. To this end, it makes a slight modification to Equation (2), which in the C&W objective determines attack success.

(4) $$\begin{array}{cc} f(\hat{x},t) & =\max\{\max_{i\neq t}\log F_{i}\left(\hat{x}\right)-\log F_{t}\left(\hat{x}\right),-\kappa \} \end{array}$$

Equation (4) modifies Equation (2) to match the assumptions of the ZOO attack. Where $F_{t}\left(x\right)$ denotes the model’s output for a given input x. In black-box settings, logits are often inaccessible, whereas softmaxed outputs may be available (i.e., $F_{t}\left(x\right)=\mathrm{softmax}\left(Z_{t}\left(x\right)\right)$). Accordingly, to obtain a proxy for the logit value from $F_{t}\left(x\right)$, ZOO uses $\log F_{t}\left(x\right)$ in place of $Z_{t}\left(x\right)$. Apart from this change, the design philosophy of Equation (4) is the same as that of Equation (2).

Now, we need to find an adversarial example that satisfies $f(\hat{x},t)$. However, in a black-box setting it is not possible to compute the gradient of $f(\hat{x},t)$ with respect to $\hat{x}$ via differentiation. Therefore, the ZOO attack numerically estimates the gradient using zeroth-order optimization.

(5) $$\begin{array}{cc} \nabla_{i}f\left(\hat{x},t\right) & =\frac{\partial f(\hat{x},t)}{\partial {\hat{x}}_{i}}\approx \frac{f\left(\hat{x}+he_{i},t\right)-f\left(\hat{x}-he_{i},t\right)}{2h} \end{array}$$

Equation (5) shows how ZOO estimates the gradient $\nabla_{i}f\left(\hat{x},t\right)$, where $e_{i}$ is the *i*-th standard basis vector in the domain of the input $\hat{x}$, and *h* is a small constant used for numerical gradient estimation. If *h* is set sufficiently small, the numerical estimate approaches the gradient computed by differentiation. However, the problem does not end there. Ideally, to estimate the gradient of $f(\hat{x},t)$ with respect to $\hat{x}$, a numerical estimate must be obtained for every dimension of the domain of $\hat{x}$. As shown in Equation (5), this process requires two feedforward evaluations for each dimension of $\hat{x}$. In other words, as the dimensionality of the input $\hat{x}$ increases, gradient estimation by Equation (5) becomes extremely costly. For this reason, ZOO employs stochastic coordinate descent, estimating gradients only for a subset of dimensions instead of all dimensions. In addition, optimization methods such as ADAM [16] or Newton’s method can be used to improve attack efficiency.

Nevertheless, gradient estimation remains highly inefficient for high-dimensional inputs. Therefore, ZOO introduces several additional heuristics. First, to estimate gradients efficiently, ZOO proposes attack-space dimension reduction. Instead of estimating perturbations in the original input dimension, it estimates them in a reduced lower-dimensional space and then upscales the result. However, estimating gradients in an excessively small dimension may fail to find a valid adversarial example. To address this, ZOO employs a hierarchical attack that expands the search space at regular iterations. Furthermore, to improve the efficiency of stochastic coordinate descent, ZOO does not select coordinates purely at random but attempts to prioritize coordinates that contribute more to the attack. To achieve this, ZOO uses importance sampling. It tracks the influence of each coordinate on the loss, treating pixels that change more under noise as more promising for the attack. Specifically, the method divides the input image into 8 × 8 blocks and computes a max-pooling map that records the maximum change within each block. The max-pooling map is then upsampled to the original image size and normalized. In this way, coordinates for gradient estimation are sampled probabilistically according to per-pixel probabilities derived from their estimated influence on the attack.

**Problem statement**. Through the design described above, the ZOO attack successfully adapts the C&W attack to black-box settings. However, we argue that ZOO is inefficient for high-dimensional, i.e., large-size, images. ❶ When performing attacks on large images, ZOO relies heavily on attack-space dimension reduction and hierarchical attack. In practice, enabling these features for large images yields adversarial examples that live in a substantially lower-dimensional space than the original image. ❷ If one disables attack-space dimension reduction and the hierarchical attack to avoid the preceding issue, ZOO becomes unacceptably slow in practice, as expected. For these reasons, we contend that more efficient and effective adversarial attacks are needed for large images than ZOO.

## 4. Fast Path-Based Zeroth Order Optimization

In this section, we describe the details of FP-ZOO, a fast patch-based zeroth order optimization attack designed to overcome the limitations of the ZOO attack. As discussed in Section 3.1, ZOO adopts a dimension-reduction strategy to perform adversarial attacks on large images efficiently; we argued that this strategy is disadvantageous for adversarial attacks where perceptibility is important. Nevertheless, in black-box settings it remains necessary to sufficiently reduce the search space to make attacks on large images efficient. Motivated by this insight, instead of resizing the original image, we partition the image into patches and propose patch-basis perturbation injection.

### 4.1. FP-ZOO Attack Algorithm

Algorithm 1 provides the pseudocode for the FP-ZOO attack. The algorithm is considerably simpler and more straightforward than ZOO. First, it initializes the accumulated change Δ as a zero tensor and sets the adversarial example $\hat{x}$ to the original image $x\in R^{H\times W\times C}$. For ease of exposition, we assume x is a squared image (i.e., H=W). Next, the sampling distribution *H* is initialized. The size of the sampling distribution *H* is determined by the image dimensions H×W×C and the patch size *P*. Concretely, *H* has *N* ($=\frac{H}{P}\times \frac{W}{P}$) elements corresponding to non-overlapping patches; each element is initialized to $\frac{1}{N}$ so that the sum of all elements equals 1. In other words, the initial sampling distribution *H* is uniform. The FP-ZOO attack then constructs the adversarial example $\hat{x}$ progressively over at most *K* iterations as follows.
**Algorithm 1:** Pseudo code for FP-ZOO attack.
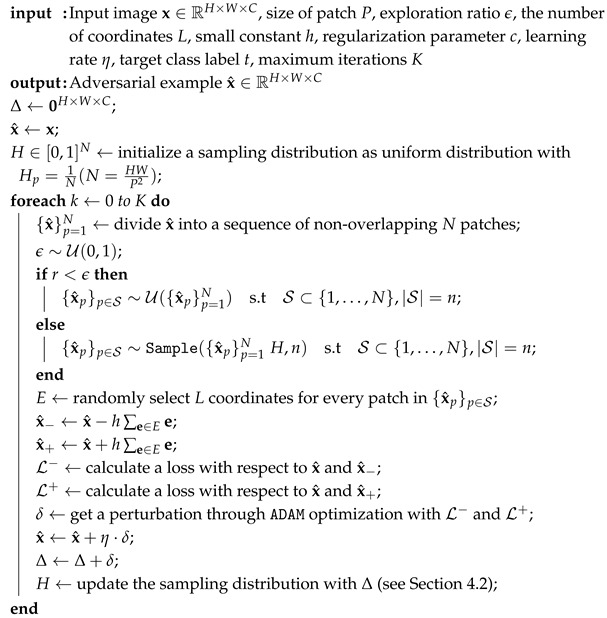


   At each iteration, the input image x is partitioned into *N* patches ${\hat{x}}_{p}\in R^{P\times P\times C}$. For ϵ-greedy scheduling, a random number r∈[0,1] is sampled. If *r* is smaller than the exploration ratio ϵ, *n* patches are selected uniformly at random to form the set ${\left\{{\hat{x}}_{p}\right\}}_{p\in S}$. Otherwise r≥ϵ, *n* patches ${\left\{{\hat{x}}_{p}\right\}}_{p\in S}$ are sampled stochastically according to the sampling distribution *H*. The purpose of ϵ-greedy scheduling is to prevent the same patches from being selected at every iteration; further details appear in Section 4.2 and Section 4.3.

For each patch in ${\left\{{\hat{x}}_{p}\right\}}_{p\in S}$, the algorithm randomly selects a set of coordinates *E* into which perturbations will be injected. For each patch ${\hat{x}}_{p}$, *L* coordinates are selected. For each coordinate $e\in {\left\{0,1\right\}}^{P\times P\times C}$, the algorithm forms ${\hat{x}}_{-}$ and ${\hat{x}}_{+}$, where *h* is a small constant (the computation of ${\hat{x}}_{-}$ and ${\hat{x}}_{+}$ in Algorithm 2 involves some abuse of notation. At the *k*-th base iteration, $\hat{x}$ has the same dimensions as the original image, H×W×C, whereas the coordinate e has dimensions P×P×C equal to the patch size. In this paper, adding or subtracting e to/from $\hat{x}$ denotes performing the corresponding operation on the specified patch). Loss values for ${\hat{x}}_{-}$ and ${\hat{x}}_{+}$ are then computed as in Equation (6).

(6) $$\begin{array}{cc} L & \leftarrow \left|\right|\hat{x}-x{\left|\right|}_{2}^{2}+c\cdot \max\left\{\max_{i\neq t}\log F_{i}\left(\hat{x}\right)-\log F_{t}\left(\hat{x}\right),-\kappa \right\} \end{array}$$

**Algorithm 2:** Pseudo code for calculating sampling distribution.**input**: Accumulated change $\Delta \in R^{H\times W\times C}$, Patch size P**output**: Sampling distribution $H\in R^{N}$${\left\{\Delta_{p}\right\}}_{p=1}^{N}\leftarrow$ divide Δ into a sequence of non-overlapping *N* patches;
$H\leftarrow \left\{\max(\right|\Delta_{p}{\left|)\right\}}_{p=1}^{N}$;
$H\leftarrow \frac{H}{\sum_{p=1}^{N}H_{p}}$;

Note that Equation (6) is the loss function for targeted attacks. By modifying this equation, we can readily derive the loss for untargeted attacks (see Equation (7)).

(7) $$\begin{array}{cc} L & \leftarrow \left|\right|\hat{x}-x{\left|\right|}_{2}^{2}+c\cdot \max\left\{\log F_{t_{0}}\left(\hat{x}\right)-\max_{i\neq t_{0}}\log F_{i}\left(\hat{x}\right),-\kappa \right\} \end{array}$$

Equations (6) and (7) are the loss functions presented by P.-Y. Chen et al. [5]. In Equation (7), $t_{0}$ denotes the target model’s initial prediction for the input image x. As shown, the targeted-attack loss function drives the logit for the target class *t* to increase, whereas the untargeted-attack loss encourages the logit of a class other than the initial prediction $t_{0}$ to increase. From the computed $L_{-}$ and $L_{+}$, we estimate the gradient with respect to the coordinates via zeroth-order optimization and obtain the optimal perturbation δ. To improve optimization efficiency, we adopt the ADAM optimizer proposed by P.-Y. Chen et al. [5]. The FP-ZOO attack updates the adversarial example by adding the perturbation δ scaled by the learning rate η to the previous $\hat{x}$. The perturbation δ is then accumulated into the accumulated change Δ. Finally, the sampling distribution *H* is updated based on the accumulated change Δ.

### 4.2. Importance Sampling

Now we examine FP-ZOO’s importance sampling in greater detail. Simply put, FP-ZOO’s importance sampling is used to sample patches, unlike the importance sampling in ZOO. As with most adversarial attacks, FP-ZOO constructs adversarial examples progressively over multiple iterations. In this process, FP-ZOO selectively injects perturbations into only a subset of the image patches for efficiency. We assume that if patches that contribute more to the attack are selected and receive perturbations more frequently, the attack will be more efficient.

Algorithm 2 shows the pseudocode for updating the sampling distribution *H* in the FP-ZOO attack. The algorithm is straightforward. First, the accumulated change Δ is partitioned into *N* patches $\Delta_{p}\in R^{P\times P\times C}$ using the patch size *P*, where *N* has the same value as in Algorithm 1. For each $\Delta_{p}$, the absolute value is computed and the maximum within the patch is taken as the representative value for that patch. In this way, we measure the largest per-patch pixel change, which reflects each patch’s contribution to the attack. Finally, the representative values are normalized to form the sampling distribution *H*.

### 4.3. ϵ-Greedy Scheduling

The reason we introduce importance sampling for patch selection is to inject perturbations primarily into patches that are meaningful for the attack. The sampling distribution *H* for importance sampling is fundamentally derived from the per-patch perturbations δ. Therefore, for a patch to obtain a nontrivial sampling probability, that patch must be selected and contribute to the attack at least once. FP-ZOO initializes the sampling distribution *H* to a uniform distribution prior to starting the iterations. Suppose a single patch is randomly selected in this initial state and receives a perturbation. The pixel-change magnitude for that patch will then be measured, causing it to appear to contribute more to the attack than other patches; in other words, it will acquire a higher sampling probability relative to the others. If this issue is left unaddressed, FP-ZOO would repeatedly select the same patch at every iteration to inject perturbations. To prevent this outcome, we adopt an ϵ-greedy scheduling strategy.

The ϵ-greedy scheduling originates from reinforcement learning [17] and uses a parameter ϵ (less than 1) to balance exploration and exploitation. In the context of FP-ZOO, exploration corresponds to selecting patches randomly. This allows patches that have not yet been used to be chosen, preventing starvation in patch selection. However, if exploration is weighted too heavily, patches that significantly contribute to the attack may be selected less frequently. Therefore, we introduce several scheduling strategies to gradually adjust the balance between exploration and exploitation.

**Constant value**. keeps ϵ fixed at the same value for all iterations, ensuring that the probability of randomly selecting patches remains constant from start to finish. This strategy is straightforward and guarantees a stable balance between random and probabilistic patch selection, but it can lead to unnecessary randomness even when the algorithm has already produced sufficiently diverse selections.

**Linear decaying**. decreases ϵ linearly as iterations progress, starting from an initial value and moving steadily toward zero. This provides a clear and predictable schedule where random patch selection is gradually reduced, allowing the process to shift from broad exploration in the early stage to more deterministic, distribution-driven choices later. However, because the decay rate is fixed, it may not perfectly match the actual needs of the patch-selection process, which can result in either too much randomness near the end or too little variation if the decay is too aggressive. Equation (8) formulates the linear decaying schedule. Where $\epsilon_{0}$ denotes the initial exploration ratio, while *K* and *k* represent the total number of iterations and the current iteration, respectively.

(8) $$\begin{array}{cc} \epsilon & \leftarrow \epsilon_{0}\times \left(1-\frac{k}{K}\right) \end{array}$$

**Exponential decaying**. reduces ϵ exponentially with the number of iterations, causing the probability of random patch selection to drop rapidly at the beginning and then taper off more slowly. This approach emphasizes strong early randomness to explore a wide variety of patches and then quickly focuses on the given probability distribution, but if the decay rate is set too high, random selection may disappear too soon and limit diversity. Equation (9) represents the exploration decaying schedule.

(9) $$\begin{array}{cc} \epsilon & \leftarrow \epsilon_{0}\times \exp\left(-\frac{k}{K}\right) \end{array}$$

**Cosine annealing**. lowers ϵ following a cosine-shaped curve across iterations, producing a smooth, non-linear decline that slows near the end and occasionally allows small surges of random patch selection. This pattern helps prevent the algorithm from getting stuck in a narrow selection pattern by reintroducing exploration opportunities, but it requires careful tuning of parameters and may prolong stabilization if re-exploration occurs too frequently. Equation (10) represents the cosine annealing scheduling strategy.

(10) $$\begin{array}{cc} \epsilon & \leftarrow \epsilon_{0}\times \frac{1}{2}\left(1+\cos\frac{k\pi }{K}\right) \end{array}$$

### 4.4. Comparison of FP-ZOO Attack and ZOO Attack

While the FP-ZOO attack shares much of its background with the ZOO attack, FP-ZOO performs attacks far more efficiently in the large-image domain (see Section 5). The primary reason for this efficiency lies in the choice of coordinates. ZOO fundamentally uses basic vectors whose dimensionality matches that of the input image as coordinates. For example, if the input image has size 32×32×3, the coordinates also have size 32×32×3. Consequently, as image size increases, the coordinate dimensionality grows and memory efficiency declines. By contrast, FP-ZOO partitions large images into much smaller patches and considers coordinates within each patch. For instance, if the input image is 224×224×3 and the patch size *P* in Algorithm 1 is 28, the original image is divided into 64 patches of size 28×28×3. Therefore, the coordinates considered by FP-ZOO have size 28×28×3. Moreover, to improve attack efficiency, FP-ZOO samples only a subset of patches for perturbation injection rather than perturbing every patch, which yields dramatically improved memory efficiency compared to ZOO. This improved memory efficiency reduces computational complexity and enables substantially faster attacks.

Another difference is importance sampling. The ZOO attack constructs a sampling distribution based on pixels (coordinates) that contribute strongly to the attack. More specifically, ZOO computes a map of each pixel’s influence on the attack, applies max pooling to produce an 8×8 sampling distribution, and then upscales this distribution to the size of the adversarial sample to obtain per-pixel probabilities. The rationale for this strategy is that, in black-box settings, efficient attacks estimate gradients by probabilistically selecting a subset of pixels rather than all pixels. Thus, instead of measuring per-pixel contribution directly, the image is divided into blocks and the sampling distribution is derived from block-level contributions. By contrast, the main difference in FP-ZOO’s importance sampling is that, whereas ZOO uses importance sampling to select coordinates, FP-ZOO uses it to sample patches into which perturbations are injected. Because FP-ZOO computes perturbations at the patch level, it is sufficient to use the maximum absolute δ within each patch. Furthermore, since the sampling distribution is used to sample patches directly, there is no need to upscale it.

## 5. Evaluations

This section presents a comprehensive evaluation of FP-ZOO’s efficiency through a series of experiments. We describe experimental details including the computing environment, hyperparameter settings, datasets, and baseline adversarial attacks. For a thorough assessment, we first establish the following three research questions (RQs).

- **RQ1**: How do key hyperparameters—such as the number of patches used for gradient estimation or the number of coordinates—affect the performance of the FP-ZOO attack?
- **RQ2**: How does the FP-ZOO attack perform compared with baseline black-box adversarial attacks across different image sizes?
- **RQ3**: How does the FP-ZOO attack perform compared with baseline white-box adversarial attacks across different image sizes?

### 5.1. Dataset Description

To evaluate the FP-ZOO attack, we use three representative image classification datasets with different resolutions and complexities: CIFAR-10 [18], CIFAR-100 [18], and ImageNet [19]. CIFAR-10 consists of 10 classes of color images sized 32×32×3 and represents a relatively simple classification task. CIFAR-100 contains images of the same size (32×32×3) but includes 100 fine-grained classes, making the classification problem comparatively more challenging. ImageNet is a large-scale, high-resolution color image dataset with 1000 classes; its complexity and scale make it suitable for evaluating the scalability of attacks in settings that resemble real-world conditions.

In all experiments except for the hyperparameter sensitivity evaluation, we use randomly selected samples from each dataset: 1000 images from CIFAR-10 and CIFAR-100, and 100 images from ImageNet. This selection allows us to systematically analyze the performance variation in the proposed attack with respect to image size and data complexity. For the untargeted attack, an attack is considered successful when the input image is misclassified into any class other than the ground-truth class. In contrast, for the targeted attack, the target class is defined according to each dataset’s characteristics. In CIFAR-10, one of the nine classes excluding the ground-truth class is randomly chosen as the target for each input image. For CIFAR-100 and ImageNet, one class other than the ground-truth class is randomly selected as the attack target for each input image.

### 5.2. Experimental Setup

**Computing environment**. All experiments were conducted on the same computing environment, consisting of an Intel(R) Core(TM) i9-11900 2.50 GHz CPU, 32 GB of RAM, and a 64-bit Ubuntu 20.04 LTS operating system. For experimental convenience, an NVIDIA GTX 3080 Titan GPU was used for hardware-accelerated computation.

**Hyper-parameter settings**. The FP-ZOO attack, like other adversarial attacks, has several hyperparameters that affect performance. Because FP-ZOO generates adversarial examples on a patch basis, we vary only a few variables related to gradient estimation (such as the number of patches and the number of coordinates); all other hyperparameters are fixed as follows. The weight variable *c* in the loss function is set to $10^{-2}$. Although ZOO recommends searching for an optimal *c* via binary search, this paper uses a constant value. The margin variable κ is set to 0, consistent with P.-Y. Chen et al. [5]. The constant *h* used for numerical gradient estimation is an important hyperparameter that substantially affects performance. In our experience, both ZOO and FP-ZOO show sensitivity of *h* to image size. In lower-resolution domains, a change of a single pixel has greater impact on the attack, so a small *h* is appropriate for fine-grained attacks; as image size increases, a larger *h* is advantageous to improve query efficiency. Therefore, we set $h=10^{-4}$ for CIFAR-10 and CIFAR-100, and $h=10^{-2}$ for ImageNet. The ADAM optimizer used for gradient estimation also requires a few hyperparameters. We set the learning rate η to $10^{-1}$, and $\beta_{1}$ and $\beta_{2}$ to 0.9 and 0.99, respectively.

**Baseline selection**. To thoroughly evaluate FP-ZOO, appropriate target models and a variety of attack techniques are required. For each dataset, the following baselines are used. For CIFAR-10 and CIFAR-100 experiments, we adopt the lightweight ResNet-20 [20] and the classical but still strong VGG-16 [21] to compare performance across different network characteristics. For ImageNet experiments, we use ResNet-50, Inception-v3 [22] (which offers diverse receptive fields), and DenseNet-121 [23] (which employs dense connectivity) to reflect large-scale, high-resolution settings. To quantitatively validate FP-ZOO’s performance, we run comparative experiments in both black-box and white-box settings. In the black-box setting, we evaluate relative performance against a range of decision- and query-based attacks, including ZOO [5] and SimBA [14]. In the white-box setting, we compare primarily with the optimization-based C&W attack [10], and include the iterative perturbation-based BIM [24] to examine differences across attack characteristics.

**Evaluation metrics**. The performance of the FP-ZOO attack is evaluated using four primary metrics. First, we measure average attack time to compare the computational efficiency required by each method to perturb the target image. We also quantify the magnitude of the perturbation by computing the $L_{2}$ distance between the original input and the attacked image, which evaluates the degree of image quality degradation. Finally, we measure attack success rate and the required average number of queries to compare each method’s ability to induce misclassification in the target model.

### 5.3. RQ1: Sensitivity to Hyper-Parameters

In this experiment, we evaluate the attack performance of the FP-ZOO attack presented in Section 4 according to its main hyperparameters. In this study, we compare the exploration ratio ϵ, the number of selected patches *n*, the number of coordinates *L*, and the performance under various scheduling strategies for ϵ. Because targeted attacks are extremely slow, we investigate the roles of multiple hyperparameters through untargeted attacks.

Table 1 presents the untargeted attack performance of the FP-ZOO attack on the CIFAR-10 and CIFAR-100 datasets with varying hyperparameters. First, we fixed *n* and *L* at 16 and 8, respectively, and used a constant scheduler while changing the exploration ratio ϵ from 0.1 to 0.5 to measure performance. The results were highly interesting. The experiments generally showed a tendency for the attack success rate to decrease as ϵ increased. In contrast, changes in ϵ had little effect on the average $L_{2}$ distance and the time required to find adversarial examples. This can be attributed to the fact that as ϵ increases, the attack attempts to inject perturbations by exploring new patches, but these newly selected patches did not meaningfully contribute to the attack. Such a pattern is consistently observed in many applications employing ϵ-greedy scheduling. Furthermore, the observation that the average $L_{2}$ distance remained similar, between 0.04 and 0.05 across all settings, indicates that the amount of perturbation injected per unit time was relatively constant. This supports the statement that smaller ϵ values tend to select patches that contribute more effectively to the attack. In terms of query efficiency, comparing only FP-ZOO attack and ZOO attack, FP-ZOO attack was slightly more query-efficient than ZOO attack. Furthermore, although not presented in the experimental results, FP-ZOO attack showed approximately 30% GPU memory usage, while ZOO attack showed approximately 70% usage. This is because in a single operation, ZOO attack loads the entire image into GPU memory, whereas FP-ZOO attack performs operations on a patch-by-patch basis.

Table 2 shows the effect of different scheduling methods on the performance of the FP-ZOO attack, where ϵ, *n*, and *L* are fixed at 0.5, 16, and 8, respectively. To clearly evaluate the impact of each scheduler, we set the initial value of ϵ to 0.5 and allowed it to be adjusted as the iterations progressed. While the linear and exponential schedulers gradually converged to zero, the cosine scheduler fluctuated over time. As observed in Table 1, the FP-ZOO attack exhibited relatively low performance with the constant scheduler, whereas experiments applying the other schedulers achieved higher attack success rates. This improvement occurs because ϵ is automatically adjusted during iterations, allowing patches that contribute more effectively to the attack to be selected more frequently. Furthermore, the cosine scheduler demonstrated better performance compared to other schedulers, which explains why exploration, rather than purely greedy patch selection, is necessary.

Finally, Table 3 presents the performance of the FP-ZOO attack with respect to the amount of injected perturbation. We set ϵ to 0.1, which produced the best performance in Table 1, fixed the number of coordinates selected per patch at 8, and used a constant scheduler. We then evaluated performance while varying the number of patches selected for perturbation injection per iteration. As shown in the table, the amount of perturbation injected at each step affected not only the attack success rate but also the $L_{2}$ distance and the time required for the attack. Injecting more perturbation per unit time led to the discovery of more adversarial examples in a shorter period. Naturally, increasing the amount of perturbation also raised the $L_{2}$ distance. In other words, injecting too much perturbation at once reduces the visual indistinguishability of the adversarial examples.

Table 4 shows the performance of FP-ZOO attack according to attack margin, regularization parameter, and learning rate. The remaining hyperparameters were set as ϵ=0.1, n=16, L=8, and constant scheduler. In this experiment, we evaluated only VGG16 as the target model and CIFAR-10 as the dataset. The reason is that previous experiments confirmed that FP-ZOO attack performs attacks sufficiently easily on datasets with various classes, and ResNet20 is a considerably robust model on CIFAR-10, so we adopted this experimental setting. As the attack margin κ increased, the attack success rate showed a noticeable decreasing trend. Attack margin is fundamentally a variable that determines how confidently the attack will be performed, and as the value increases, the logit for the target model’s initial prediction on a given input must become lower. The regularization parameter c represents the degree of reflection of attack objectives in the loss function. That is, as *c* increases, it focuses more on successful attacks rather than $L_{2}$ distance. Consequently, as *c* increases, the attack success rate improves and adversarial examples are generated more easily, while the $L_{2}$ distance shows a slightly increasing trend. Finally, the learning rate η is involved in ADAM optimization calculating gradients during the FP-ZOO attack. Fundamentally, as η decreases, the intensity of perturbations injected at once weakens, making it more difficult for FP-ZOO attack to search for adversarial examples.

### 5.4. RQ2: Comparative Study with Other Black-Box Attacks

We compare the FP-ZOO attack with other representative black-box attacks. The baselines for this experiment are the ZOO attack and SimBA. ZOO is a direct predecessor of FP-ZOO, and SimBA is known as a simple yet effective attack. To ensure a fair comparison between FP-ZOO and ZOO, we attempted to match hyperparameter settings. For example, when ZOO selects 128 coordinates per iteration, we configured FP-ZOO with n=16 and L=8 so that 128 coordinates are selected at once. Note that, for a fair comparison, binary search and image resizing are disabled in the ZOO attack.

Table 5 shows the untargeted attack performance of FP-ZOO, ZOO, and SimBA on the CIFAR-10 and CIFAR-100 datasets. FP-ZOO achieved higher attack success rates than ZOO in all cases. FP-ZOO also required less time to reach successful attacks compared to ZOO, which follows from the patch-based design of FP-ZOO improving computational efficiency as described in Section 4. In contrast, ZOO produced better $L_{2}$ distances than FP-ZOO, likely because ZOO’s finer-grained gradient estimation avoids unnecessary perturbation. SimBA outperformed the other black-box attacks overall; unlike FP-ZOO and ZOO, SimBA accumulates perturbations that affect the model logits for a given input instead of estimating gradients, enabling more efficient attacks at the cost of producing relatively coarser perturbations.

Table 6 compares the targeted attack performance of FP-ZOO, ZOO, and SimBA on the CIFAR-10 and CIFAR-100 datasets. This result is also particularly interesting. Under the targeted attack setting, FP-ZOO demonstrated lower performance than ZOO under the same conditions, while SimBA continued to show high performance. We analyze the results focusing on FP-ZOO and ZOO. As mentioned earlier, the FP-ZOO attack is designed with a patch-based structure that prioritizes computational efficiency, which makes its gradient estimation less fine-grained than that of ZOO. More specifically, in FP-ZOO, when a patch is selected in an iteration, *L* perturbations are injected within that patch even if some of them do not contribute meaningfully to the attack. However, targeted attacks have narrower objectives than untargeted attacks and therefore require more precise perturbation injection for a given image. This explains why FP-ZOO performs worse than ZOO in targeted attacks. In terms of $L_{2}$ distance and the time required for successful attacks, FP-ZOO also struggled more to find adversarial examples.

Table 7 compares the performance of black-box attacks in both untargeted and targeted settings on the ImageNet dataset. In this experiment, the hyperparameters of each attack were set identically to those in Table 6. Because the ImageNet dataset is more complex than CIFAR-10 and CIFAR-100, the experimental configuration from Table 6 may not be entirely appropriate when considering practical performance. Reducing the attack intensity, however, helps to better highlight the characteristics of each attack. As expected, all attacks found it more difficult to generate adversarial examples compared to the CIFAR-10 and CIFAR-100 experiments, as indicated by the lower attack success rates and higher average times. In the untargeted setting, FP-ZOO generated adversarial examples significantly faster than ZOO and achieved a higher attack success rate, reaffirming that the patch-based design of FP-ZOO enables more efficient computation for larger images. In contrast, both FP-ZOO and ZOO performed poorly in targeted attacks, with FP-ZOO showing even lower performance in terms of average time. This again stems from FP-ZOO’s coarse gradient estimation. Meanwhile, SimBA maintained stable performance compared to the other two attacks.

Table 8 compares the performance of FP-ZOO attack and ZOO attack in untargeted attacks against vision models with adversarial training applied. In this experiment, ResNet-50 [25] and Vision Transformer (ViT) [26] were employed as target models. According to the experimental results, compared to Table 7, both FP-ZOO attack and ZOO attack showed decreased attack success rates against models with adversarial training applied. Furthermore, it was observed that the time and queries required to search for adversarial examples increased noticeably. This indicates that adversarial training is indeed effective as a defense method against adversarial attacks. Nevertheless, these experimental results suggest that additional countermeasures are required, as adversarial training can be neutralized if a sufficient window of attack opportunity is provided.

### 5.5. RQ3: Comparative Study with White-Box Attacks

Finally, we compare FP-ZOO with several white-box attacks. Because FP-ZOO is designed as a black-box attack, it is inherently less efficient than white-box attacks. Nevertheless, we conducted this comparison experiment to assess how much performance FP-ZOO sacrifices compared to white-box attacks. In this experiment, BIM and C&W attacks were selected as the baseline white-box attacks. BIM is one of the simplest white-box attacks, while C&W is a direct predecessor of FP-ZOO and ZOO, making it a suitable baseline.

Table 9 compares the performance of several white-box attacks and the FP-ZOO attack under both untargeted and targeted attack settings. The FP-ZOO attack was configured with ϵ=0.1, n=16, L=8, and a constant scheduler. The BIM attack was set with a maximum perturbation of 0.3 and a step size of 0.01. The C&W attack was configured with a loss weight variable $c=10^{-2}$, a margin variable κ=0, and a learning rate $\eta =10^{-2}$. As shown in the table, FP-ZOO generally performed worse than the white-box attacks. Although BIM is one of the simplest methods, it demonstrated stable and high performance because it directly computes the gradient of the loss function with respect to the input image and derives the most effective perturbation for the attack. In contrast, while C&W is also a white-box attack, it adopts a fundamentally different approach. C&W maps noise in the tanh space to the input image domain and differentiates the loss with respect to this transformed input to generate adversarial examples. This strategy enables C&W to produce visually smooth adversarial examples even at high attack intensities, although it results in a slightly higher $L_{2}$ distance compared to the original image. Compared to these baseline white-box attacks, FP-ZOO shows lower performance in terms of attack success rate, average $L_{2}$ distance, and average time, with a particularly noticeable weakness in targeted attacks. Nevertheless, FP-ZOO exhibits a relatively competitive attack success rate and a lower $L_{2}$ distance in untargeted attacks.

### 5.6. Visual Comparison

Finally, we conduct a visual analysis of the adversarial examples generated by the FP-ZOO attack. To achieve this, we compare the original images, adversarial examples, perturbations, and sampling probabilities from the CIFAR-10, CIFAR-100, and ImageNet datasets.

**Figure 1:**
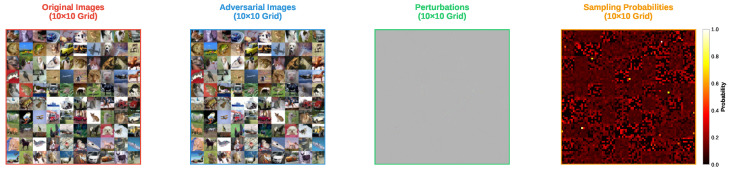
Visualization of adversarial examples from FP-ZOO attack targeting ResNet20 in CIFAR-10.

**Figure 2:**
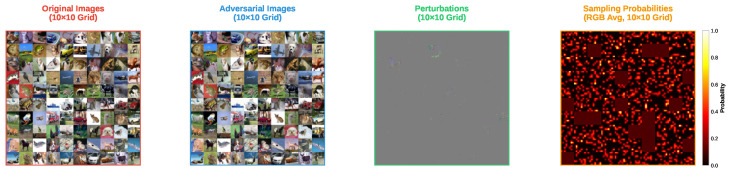
Visualization of adversarial example from ZOO attack targeting ResNet20 in CIFAR-10.

Figure 1 and Figure 2 present the adversarial examples and perturbations for 100 randomly selected images from the CIFAR-10 dataset, respectively. In addition, the rightmost subfigure in both figures shows the sampling probabilities when the adversarial examples were found. However, the interpretation of these probabilities differs between the FP-ZOO attack and the ZOO attack.

In the FP-ZOO attack, the sampling probability distribution represents the probability of selecting patches. Specifically, each image is divided into 8×8 patches, and the probabilities indicate the likelihood of each patch being chosen. In contrast, in the ZOO attack, the probability distribution is used to select coordinates for coordinate descent. As mentioned earlier, the ZOO attack typically employs image rescaling for computational efficiency, but this feature was disabled in this experiment when calculating sampling probabilities.

As shown in the figures, both FP-ZOO and ZOO attacks inject very small perturbations into the images, making it impossible to visually distinguish the adversarial examples from the originals. However, the perturbation patterns differ notably between the two attacks. The ZOO attack injects perturbations mainly into pixels that have a high contribution to the attack’s success, resulting in locally concentrated perturbations. In contrast, the FP-ZOO attack stochastically selects patches and injects perturbations randomly within each patch, leading to more evenly distributed perturbations across the image.

**Figure 3:**
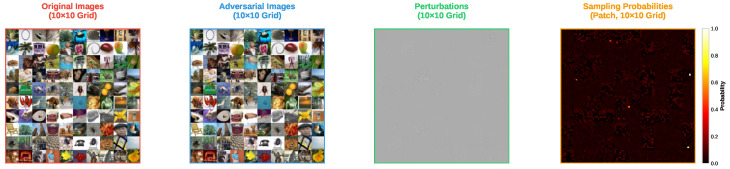
Visualization of adversarial examples from FP-ZOO attack targeting ResNet20 in CIFAR-100.

**Figure 4:**
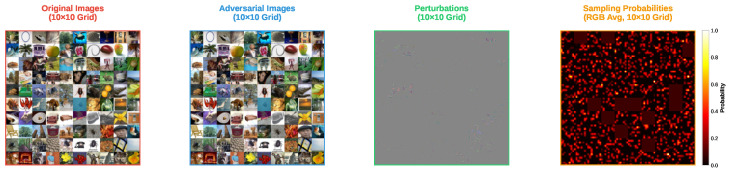
Visualization of adversarial examples from ZOO attack targeting ResNet20 in CIFAR-100.

Figure 3 and Figure 4 show the adversarial examples generated by the FP-ZOO attack and the ZOO attack on CIFAR-100, respectively. Because CIFAR-100 is essentially a refinement of CIFAR-10 labels while sharing the same image modality, the results of this experiment are similar to the previous one. One notable difference is that the sampling distributions measured in this experiment are relatively sparser than in the previous experiment. This is because all attacks produced adversarial examples for ResNet20 on CIFAR-100 more quickly than on CIFAR-10. More specifically, since the FP-ZOO attack found adversarial examples rapidly, many patches were not selected, and in the ZOO attack many pixels remained unselected. In other words, the ResNet20 trained on CIFAR-100 was more vulnerable than the one trained on CIFAR-10, a phenomenon often observed in models with a larger number of classes. As the number of classes increases, the amount of training data per class decreases, which can make decision boundaries between classes less robust.

**Figure 5:**
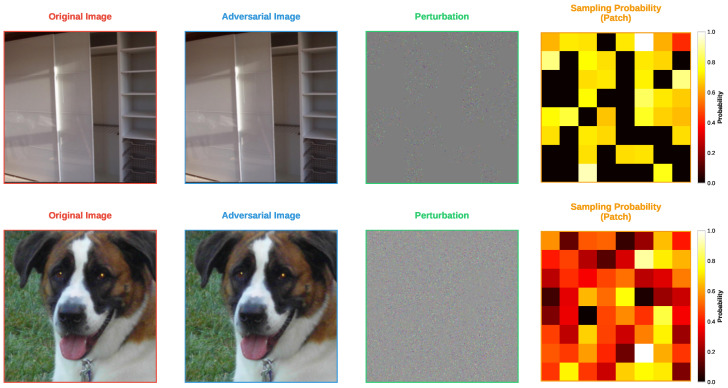
Visualization of adversarial examples from FP-ZOO attack targeting ResNet50 in ImageNet.

**Figure 6:**
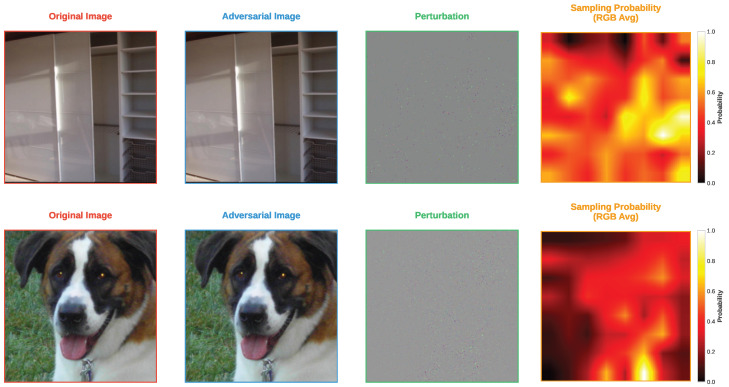
Visualization of adversarial examples from ZOO attack targeting ResNet50 in ImageNet.

Finally, Figure 5 and Figure 6 show the adversarial examples generated by the FP-ZOO attack and the ZOO attack on ImageNet, respectively. Because the images are larger, the characteristics of each attack are more clearly distinguishable compared to CIFAR-10 and CIFAR-100. In both attacks, the generated adversarial examples are visually indistinguishable from the original images. However, clear differences can be observed in the perturbation patterns. The perturbations of the adversarial examples generated by the ZOO attack exhibit more pronounced pixel-level tendencies, whereas those of the FP-ZOO attack appear completely random at the pixel level but show a pattern at the patch level.

An interesting observation is that the pixel-level sampling distribution of the ZOO attack and the patch-level sampling distribution of the FP-ZOO attack share some similarities. This feature is particularly noticeable in the second row of each figure, where the locations of patches with high sampling probabilities in the FP-ZOO attack roughly correspond to the locations of pixels with high probabilities in the ZOO attack. Taken together with the previous experiments, these results indicate that the FP-ZOO attack performs stochastic selection at the patch level and injects perturbations randomly within each patch, resulting in relatively coarse adversarial examples. Therefore, the degree of fineness of the FP-ZOO attack should be adjusted by appropriately tuning its hyperparameters.

Table 10 compares the perceptual similarity of adversarial examples generated by FP-ZOO attack and ZOO attack in untargeted attacks. We adopt the structural similarity index measure (SSIM) and peak signal-to-noise ratio (PSNR) as evaluation metrics for measuring perceptual similarity. SSIM models how the human visual system perceives structural information in images, where values closer to 1 indicate that adversarial examples are more similar to the original images. PSNR measures the quality loss between original and processed images, where higher values indicate higher-quality adversarial examples. As shown in Table 10, while the performance of FP-ZOO attack was measured to be sufficiently good, overall the quality of adversarial examples generated by ZOO attack was found to be higher. There are two reasons for this. First, as mentioned previously, FP-ZOO attack injects perturbations into images more coarsely compared to ZOO attack. Consequently, relatively more unnecessary perturbations are injected compared to ZOO attack, which degrades perceptual similarity. Second, the reason lies in the high attack success rate of FP-ZOO attack. FP-ZOO attack demonstrated superior performance compared to ZOO attack in untargeted attacks, which means it generated adversarial examples that ZOO attack failed to find, and these examples likely required more perturbations as they were more difficult to discover. Consequently, this lowers the average SSIM and PSNR metrics of adversarial examples generated by FP-ZOO attack.

## 6. Discussions

In this section, we discuss the FP-ZOO attack based on the evaluation results presented in Section 5. FP-ZOO was designed as an improvement over ZOO and has the following clear advantages. ➀ The patch-based perturbation injection of FP-ZOO reduces the size of the coordinate vector during iterations, enabling more efficient computation. This is important because ZOO inherently generates many coordinate vectors that are the same size as the input image, which increases memory complexity and degrades computational speed as image size grows. By contrast, FP-ZOO divides the image into patches and adopts coordinate vectors sized to the patches, allowing much more efficient computation. ➁ FP-ZOO can generate adversarial examples effectively without downscaling the input image. To mitigate the computational inefficiency noted above, ZOO uses image resizing. We observed that for models using large input images, ZOO’s image resizing contributes more to success than the perturbation injection. Because FP-ZOO injects perturbations on a patch basis, it can attack at the original image resolution without resizing. ➂ The patch-based attack approach used by FP-ZOO is generally applicable and can be adopted by other types of attacks beyond ZOO. Furthermore, the patch-based approach has value because it can be transferred not only to black-box attacks but also to white-box attacks.

FP-ZOO attack, despite having the aforementioned clear advantages, also exhibits several distinct limitations revealed through experiments. ❶ FP-ZOO attack generates more perturbations than necessary. This explains the higher $L_{2}$ distance observed in Section 5 when compared with the ZOO attack. While FP-ZOO is computationally efficient due to its patch-based perturbation injection, each patch must contain a fixed number (*L*) of perturbations. As a result, noise that does not contribute to attack success may be generated, and the accumulation of such noise increases the $L_{2}$ distance. Therefore, it is necessary to adjust the hyperparameters of FP-ZOO appropriately to maintain the visual indistinguishability of adversarial examples. Another possible approach is to adjust the number of coordinates selected per patch. While the FP-ZOO attack stochastically selects patches that are effective for the attack, it randomly selects coordinates within each patch. Extending this approach, the amount of perturbations could be controlled by selecting more coordinates from patches that are more effective for the attack and fewer coordinates from less critical patches. ❷ FP-ZOO attack has a clear limitation in targeted attacks. Because targeted attacks have narrower objectives than untargeted ones, they require finer perturbations. However, due to the issue mentioned above, FP-ZOO inherently produces coarse perturbations, which leads to its lower success rate in targeted attack settings. From this, a strategy to overcome the low performance of targeted attacks naturally emerges. Instead of randomly selecting coordinates within the patch, which is the current approach of the FP-ZOO attack, if we specifically identify coordinates within the patch that are actually effective for the attack and inject perturbations at those locations, it would be possible to achieve sufficiently effective attacks even for targeted attacks.

Additionally, we conducted large-scale experiments comparing the FP-ZOO attack with several baseline attack techniques. However, there remain important aspects that have not yet been addressed. First, in our experiments, we measured the average number of queries required for adversarial attacks to generate adversarial examples under each experimental setting. In real-world scenarios, the window of opportunity for attacking a target model is highly limited, necessitating the maximization of attack performance under restricted queries. However, we did not address this aspect in our experiments. Nevertheless, it is encouraging that the FP-ZOO attack was faster and more query-efficient compared to the ZOO attack in untargeted attacks. In contrast, it was confirmed that improvement is still needed in targeted attacks. Furthermore, we did not include the latest state-of-the-art adversarial attack methods as baselines. While this limits the strength of our comparative evaluation, it would require another large-scale experimental study. Therefore, we leave these two experimental limitations as open problems.

The whole point of this study is to efficiently perform attacks against vision models in black-box environments, and consequently to improve the robustness and reliability of the models. There can be several approaches to achieve this. First, the FP-ZOO attack is fundamentally performed based on changes in the model’s logits for input images according to perturbations. Therefore, if a vision model outputs classification results instead of logits for a given input, it can be used more safely against various logits-based attacks including the FP-ZOO attack. Second, a more robust model can be built through preprocessing before the input is passed to the model [27,28]. Perturbations are a type of noise that is very delicately designed with a specific purpose. Therefore, if preprocessing such as image scaling or filtering is applied before adversarial examples are input to the model, the effect of adversarial examples can be neutralized. Finally, adversarial training can be considered [29,30]. Adversarial training includes adversarial examples in the training data to train the model. By doing so, the decision boundary that the model learns becomes smoother and the model becomes more robust against attacks.

## 7. Conclusions

In this paper, we propose FP-ZOO, a patch-based fast zeroth-order optimization adversarial attack. Unlike ZOO, FP-ZOO can perform attacks on large images without downscaling and enables efficient computation by injecting perturbations at the patch level. The effectiveness of this approach was demonstrated through extensive large-scale experiments on datasets of various sizes. Although FP-ZOO showed several advantages in large-image domains, experiments revealed some limitations. We discussed the strengths and weaknesses of FP-ZOO candidly. In particular, we expect that the limitations of FP-ZOO can be mitigated by adaptively selecting coordinates using importance sampling. Accordingly, we plan to investigate more efficient patch-based black-box attacks for targeted settings in future work. To emphasize once again, this study effectively discovers vulnerabilities of vision models from an adversary’s perspective. Such research supports the safe operation of AI systems in various domains where AI adoption is being pursued, such as autonomous driving, healthcare, and smart factories. 

## Figures and Tables

**Table 1:** Performance evaluations on exploration ratio for untargeted attack.

Settings				Dataset	Target Model	Success Rate	Avg. $L_{2}$	Avg. Time	Avg. #Queries
ϵ	n	L	Scheduler						
0.1	16	8	constant	CIFAR-10	VGG16	94.8%	0.05±0.03	25.74 s ± 32.39	1801.82±2267.3
0.1	16	8	constant	CIFAR-10	ResNet20	83.2%	0.05±0.03	4.46 s ± 13.17	312.63±921.9
0.1	16	8	constant	CIFAR-100	VGG16	97.6%	0.05±0.03	6.55 s ± 16.22	458.57±1135.4
0.1	16	8	constant	CIFAR-100	ResNet20	99.2%	0.04±0.02	0.31 s ± 3.16	21.78±221.2
0.25	16	8	constant	CIFAR-10	VGG16	93.7%	0.05±0.03	26.09 s ± 49.42	21.78±221.2
0.25	16	8	constant	CIFAR-10	ResNet20	82.9%	0.05±0.03	4.21 s ± 12.33	294.76±863.1
0.25	16	8	constant	CIFAR-100	VGG16	97.4%	0.04±0.02	6.56 s ± 16.01	459.21±1120.7
0.25	16	8	constant	CIFAR-100	ResNet20	99.2%	0.04±0.02	0.29 s ± 2.59	20.39±181.3
0.5	16	8	constant	CIFAR-10	VGG16	91.7%	0.05±0.03	26.5 s ± 34.4	1855.29±2408.2
0.5	16	8	constant	CIFAR-10	ResNet20	82.8%	0.05±0.03	4.42 s ± 12.65	309.47±885.5
0.5	16	8	constant	CIFAR-100	VGG16	97.1%	0.04±0.02	6.86 s ± 16.28	480.25±1139.6
0.5	16	8	constant	CIFAR-100	ResNet20	99.2%	0.04±0.02	0.29 s ± 2.6	20.3±182.5

**Table 2:** Performance evaluations on scheduling method for untargeted attack.

Settings				Dataset	Target Model	Success Rate	Avg. $L_{2}$	Avg. Time	Avg. #Queries
ϵ	n	L	Scheduler						
0.5	16	8	constant	CIFAR-10	VGG16	91.7%	0.05 ± 0.03	26.5 s ± 34.4	1855.29±2408.2
0.5	16	8	constant	CIFAR-10	ResNet20	82.8%	0.05±0.03	4.42 s ± 12.65	309.47±885.5
0.5	16	8	constant	CIFAR-100	VGG16	97.1%	0.04±0.02	6.86 s ± 16.28	480.25±1139.6
0.5	16	8	constant	CIFAR-100	ResNet20	99.2%	0.04±0.02	0.29 s ± 2.6	20.3±182.5
0.5	16	8	linear	CIFAR-10	VGG16	93.5%	0.05±0.03	26.77 s ± 34.08	1873.9±2385.6
0.5	16	8	linear	CIFAR-10	ResNet20	83.2%	0.05±0.03	4.86 s ± 14.4	340.2±1008.9
0.5	16	8	linear	CIFAR-100	VGG16	97.3%	0.04±0.02	6.91 s ± 16.46	483.7±1152.2
0.5	16	8	linear	CIFAR-100	ResNet20	99.2%	0.03±0.02	0.31 s ± 3.18	21.7±222.6
0.5	16	8	exponential	CIFAR-10	VGG16	93.2%	0.05±0.03	26.89 s ± 34.38	1882.3±2406.6
0.5	16	8	exponential	CIFAR-10	ResNet20	83.2%	0.05±0.03	4.78 s ± 13.74	334.6±961.8
0.5	16	8	exponential	CIFAR-100	VGG16	97.4%	0.05±0.03	7.08 s ± 17.08	495.6±1195.6
0.5	16	8	exponential	CIFAR-100	ResNet20	99.2%	0.04±0.02	0.27 s ± 2.21	18.9±154.7
0.5	16	8	cosine	CIFAR-10	VGG16	93.7%	0.05±0.03	26.48 s ± 34.83	1853.6±2438.1
0.5	16	8	cosine	CIFAR-10	ResNet20	83.2%	0.05±0.03	5.29 s ± 16.91	391.3±1183.7
0.5	16	8	cosine	CIFAR-100	VGG16	97.4%	0.04±0.02	7.01 s ± 16.76	490.7±1173.2
0.5	16	8	cosine	CIFAR-100	ResNet20	99.2%	0.04±0.02	0.29 s ± 2.61	20.3±182.7

**Table 3:** Performance evaluations according to the amount of injected perturbation for untargeted attack.

Settings				Dataset	Target Model	Success Rate	Avg. $L_{2}$	Avg. Time	Avg. #Queries
ϵ	n	L	Scheduler						
0.1	16	8	constant	CIFAR-10	VGG16	94.8%	0.05±0.03	25.74 s ± 32.39	1801.82±2267.3
0.1	16	8	constant	CIFAR-10	ResNet20	83.2%	0.05±0.03	4.46 s ± 13.17	312.63±921.9
0.1	16	8	constant	CIFAR-100	VGG16	97.6%	0.05±0.03	6.55 s ± 16.22	458.57±1135.4
0.1	16	8	constant	CIFAR-100	ResNet20	99.2%	0.04±0.02	0.31 s ± 3.16	21.78±221.2
0.1	32	8	constant	CIFAR-10	VGG16	98.6%	0.06±0.03	24.22 s ± 33.85	968.8±1354.8
0.1	32	8	constant	CIFAR-10	ResNet20	85.9%	0.07±0.03	4.69 s ± 14.23	187.6±569.2
0.1	32	8	constant	CIFAR-100	VGG16	99.2%	0.06±0.03	5.57 s ± 17.61	222.8±704.4
0.1	32	8	constant	CIFAR-100	ResNet20	99.4%	0.05±0.03	0.29 s ± 3.85	11.6±154.7

**Table 4:** Performance evaluations according to attack margin, regularization parameter, and learning rate in untargeted attack.

Settings			Success Rate	Avg. $L_{2}$	Avg. Time	Avg. #Queries
κ	c	η				
0	0.01	0.1	94.8%	0.05±0.03	25.74 s ± 32.39	1801.82±2267.3
0.1	0.01	0.1	91.3%	0.04±0.01	33.65 s ± 41.74	2457.36±2981.8
0.2	0.01	0.1	73.6%	0.04±0.01	39.95 s ± 44.78	2804.7±3232.5
0	0.1	0.1	100%	0.06±0.01	6.01 s ± 6.83	426.35±486.15
0	0.5	0.1	100%	0.06±0.02	1.75 s ± 1.84	123.65±131.25
0	0.01	0.01	54.7%	0.01±0.01	27.38 s ± 40.77	1968.6±2936.88
0	0.01	0.001	32.4%	0.01±0.01	67.45 s ± 55.28	3784.1±4781.36

**Table 5:** Comparative evaluations of various black-box attacks for untargeted attacks.

**Attack**	**Settings**				**Dataset**	**Target Model**	**Success Rate**	**Avg.** $L_{2}$	**Avg. Time**	**Avg. #Queries**
	ϵ	n	L	**Scheduler**						
FP-ZOO	0.1	16	8	constant	CIFAR-10	VGG16	94.8%	0.05±0.03	25.74 s ± 32.39	1801.82±2267.3
	0.1	16	8	constant	CIFAR-10	ResNet20	83.2%	0.05±0.03	4.46 s ± 13.17	312.63±921.9
	0.1	16	8	constant	CIFAR-100	VGG16	97.6%	0.05±0.03	6.55 s ± 16.22	458.57±1135.4
	0.1	16	8	constant	CIFAR-100	ResNet20	99.2%	0.04±0.02	0.31 s ± 3.16	21.78±221.2
	0.1	32	8	constant	CIFAR-10	VGG16	98.6%	0.06±0.03	24.22 s ± 33.85	968.8±1354.8
	0.1	32	8	constant	CIFAR-10	ResNet20	85.9%	0.07±0.03	4.69 s ± 14.23	187.6±569.2
	0.1	32	8	constant	CIFAR-100	VGG16	99.1%	0.06±0.03	5.57 s ± 17.61	222.8±704.4
	0.1	32	8	constant	CIFAR-100	ResNet20	99.4%	0.05±0.03	0.29 s ± 3.85	11.6±154.7
**Attack**	**The number of coordinates**				**Dataset**	**Target model**	**Success rate**	**Avg. $L_{2}$**	**Avg. Time**	**Avg. #Queries**
ZOO	128				CIFAR-10	VGG16	76.7%	0.02±0.01	28.66±43.26	1862.94±2811.9
	128				CIFAR-10	ResNet20	77.0%	0.02±0.01	8.12 s ± 6.85	527.81±445.25
	128				CIFAR-100	VGG16	93.4%	0.02±0.01	10.57 s ± 49.22	687.05±3199.3
	128				CIFAR-100	ResNet20	99.0%	0.02±0.01	1.11 s ± 0.62	75.15±40.3
	256				CIFAR-10	VGG16	85.0%	0.02±0.01	27.98 s ± 60.46	1119.25±2417.4
	256				CIFAR-10	ResNet20	77.1%	0.02±0.01	7.60 s ± 4.81	304.97±192.4
	256				CIFAR-100	VGG16	94.5%	0.02±0.01	9.08 s ± 23.82	363.21±952.8
	256				CIFAR-100	ResNet20	99.0%	0.02±0.01	1.10 s ± 0.51	44.37±20.4
**Attack**	**Step size**				**Dataset**	**Target model**	**Success rate**	**Avg. $L_{2}$**	**Avg. Time**	**Avg. #Queries**
SimBA	0.2				CIFAR-10	VGG16	100%	0.08±0.05	1.70 s ± 2.05	449.51±533.7
	0.2				CIFAR-10	ResNet20	95.2%	0.05±0.03	0.8 s ± 0.63	208.37±163.8
	0.2				CIFAR-100	VGG16	100%	0.04±0.02	0.73 s ± 3.26	189.82±847.6
	0.2				CIFAR-100	ResNet20	99.8%	0.03±0.02	0.42 s ± 0.53	109.27±137.8

**Table 6:** Comparative evaluations of various black-box attacks for targeted attacks.

**Attack**	**Settings**				**Dataset**	**Target Model**	**Success Rate**	**Avg.** $L_{2}$	**Avg. Time**	**Avg. #Queries**
	ϵ	n	L	**Scheduler**						
FP-ZOO	0.1	16	8	constant	CIFAR-10	VGG16	55.3%	0.30±0.18	53.19 s ± 78.51	3723.36±5195.7
	0.1	16	8	constant	CIFAR-10	ResNet20	79.5%	0.33±0.19	47.00 s ± 65.17	3290.12±4561.9
	0.1	16	8	constant	CIFAR-100	VGG16	54.8%	0.39±0.21	68.20 s ± 98.73	4774.65±6911.1
	0.1	16	8	constant	CIFAR-100	ResNet20	89.1%	0.47±0.24	32.84 s ± 48.10	2298.83±3367.5
**Attack**	**The number of coordinates**				**Dataset**	**Target model**	**Success rate**	**Avg. $L_{2}$**	**Avg. Time**	**Avg. #Queries**
ZOO	128				CIFAR-10	VGG16	64.0%	0.25±0.16	44.63 s ± 65.98	2900.95±4288.7
	128				CIFAR-10	ResNet20	67.4%	0.19±0.12	42.58 s ± 61.22	2767.73±3979.3
	128				CIFAR-100	VGG16	73.0%	0.33±0.23	57.23±80.95	3719.95±5261.75
	128				CIFAR-100	ResNet20	98.9%	0.15±0.08	26.32 s ± 39.32	1710.82±2555.8
**Attack**	**Step size**				**Dataset**	**Target model**	**Success rate**	**Avg. $L_{2}$**	**Avg. Time**	**Avg. #Queries**
SimBA	0.2				CIFAR-10	VGG16	97.8%	0.12±0.07	5.08 s ± 10.71	1320.85±2784.6
	0.2				CIFAR-10	ResNet20	100%	0.07±0.03	1.34 s ± 7.67	348.47±1994.2
	0.2				CIFAR-100	VGG16	98.6%	0.13±0.07	6.99 s ± 15.28	1817.43±3972.8
	0.2				CIFAR-100	ResNet20	100%	0.07±0.03	1.89 s ± 8.41	491.46±2186.6

**Table 7:** Comparative evaluations on ImageNet in both untargeted and targeted attacks.

Attack	Target Model	Untargeted Attack				Targeted Attack			
		**Success Rate**	Avg. $L_{2}$	Avg. Time	Avg. #Queries	Success Rate	Avg. $L_{2}$	Avg. Time	Avg. #Queries
FP-ZOO	ResNet50	97.0%	0.02	16.62 s	551.3	10%	0.04	212.08 s	4453.68
	Inception v3	79.0%	0.02	57.62 s	1358.07	2%	0.01	284.04 s	5964.84
	DenseNet121	100%	0.01	32.87 s	833.28	20%	0.09	296.94 s	6235.74
ZOO	ResNet50	95.0%	0.01	27.50 s	498.6	11%	0.04	200.68 s	6362.4
	Inception v3	76.0%	0.01	64.67 s	1728.6	2%	0.01	246.46 s	8521.2
	DenseNet121	100%	0.01	39.68 s	986.1	20%	0.05	267.53 s	8908.2
SimBA	ResNet50	100%	0.01	9.87 s	888.3	74%	0.04	115.53 s	5755.2
	Inception v3	92%	0.01	29.42 s	2647.8	55%	0.05	238.04 s	8168.4
	DenseNet121	100%	0.01	12.92 s	1162.8	82%	0.04	299.50 s	9137.6

**Table 8:** Performance evaluations on models with defense.

Setting	Target Model	Success Rate	Avg. $L_{2}$	Avg. Time	Avg. #Queries
FP-ZOO	ResNet50	94.74%	0.04	61.42 s	2072.47
	ViT	91.46%	0.02	192.4 s	3082.88
ZOO	ResNet50	93.35%	0.03	367.3 s	2235.61
	ViT	90.84%	0.01	851.5 s	3267.05

**Table 9:** Comparative evaluations of FP-ZOO attack with white-box attacks.

Attack	Dataset	Target Model	Untargeted Attack			Targeted Attack		
			Success Rate	Avg. $L_{2}$	Avg. Time	Success Rate	Avg. $L_{2}$	Avg. Time
FP-ZOO	CIFAR-10	VGG16	94.8%	0.05±0.03	25.74 s ± 32.39	55.3%	0.30±0.18	53.19 s ± 78.51
	CIFAR-10	ResNet20	83.2%	0.05±0.03	4.46 s ± 13.17	79.5%	0.33±0.19	47.00 s ± 65.17
	CIFAR-100	VGG16	97.6%	0.05±0.03	6.55 s ± 16.22	54.8%	0.39±0.21	68.20 s ± 98.73
	CIFAR-100	ResNet20	99.2%	0.04±0.02	0.31 s ± 3.16	89.1%	0.47±0.24	32.84 s ± 48.10
	ImageNet	ResNet50	97.0%	0.02	16.62 s	10%	0.04	212.08 s
	ImageNet	Inception v3	79.0%	0.02	57.62 s	2%	0.01	284.04 s
	ImageNet	DenseNet121	100%	0.01	32.87 s	20%	0.09	296.94 s
BIM	CIFAR-10	VGG16	100%	0.03±0.01	0.03 s ± 0.07	98.6%	0.08±0.05	0.59 s ± 0.46
	CIFAR-10	ResNet20	100%	0.01±0.01	0.02 s ± 0.07	100%	0.02±0.01	0.03 s ± 0.07
	CIFAR-100	VGG16	100%	0.01±0.01	0.02 s ± 0.07	100%	0.06±0.03	0.33 s ± 0.12
	CIFAR-100	ResNet20	100%	0.01±0.01	0.02 s ± 0.07	100%	0.02±0.01	0.03 s ± 0.07
	ImageNet	ResNet50	100%	0.01	0.03 s	100%	0.02	0.07 s
	ImageNet	Inception v3	100%	0.01	0.06 s	100%	0.03	0.29 s
	ImageNet	DenseNet121	100%	0.01	0.06 s	100%	0.01	0.15 s
C&W	CIFAR-10	VGG16	100%	0.72±0.37	0.05 s ± 0.03	97.1%	0.72±0.24	0.71 s ± 0.13
	CIFAR-10	ResNet20	100%	0.72±0.37	0.03 s ± 0.02	99.7%	0.72±0.24	0.17 s ± 0.08
	CIFAR-100	VGG16	100%	0.76±0.38	0.02 s ± 0.01	93.9%	0.74±0.25	1.08 s ± 0.27
	CIFAR-100	ResNet20	100%	0.76±0.38	0.02 s ± 0.01	97.9%	0.75±0.25	0.37 s ± 0.12
	ImageNet	ResNet50	100%	0.91	0.05 s	99%	0.91	0.41 s
	ImageNet	Inception v3	100%	0.91	0.14 s	99%	0.91	1.95 s
	ImageNet	DenseNet121	100%	0.91	0.13 s	99%	0.91	0.75 s

**Table 10:** Comparison for perceptual similarity between FP-ZOO attack and ZOO attack.

Setting	Dataset	Target Model	SSIM	PSNR
FP-ZOO	CIFAR-10	VGG16	0.9872±0.01	39.0716±2.84
	CIFAR-10	ResNet20	0.9893±0.01	38.5179±3.29
	CIFAR-100	VGG16	0.9861±0.01	37.4322±2.84
	CIFAR-100	ResNet20	0.9897±0.01	38.4767±4.37
	ImageNet	ResNet50	0.9855±0.01	46.7347±4.43
	ImageNet	Inception v3	0.9786±0.01	43.6731±2.69
	ImageNet	DenseNet121	0.9923±0.01	48.2188±2.27
ZOO	CIFAR-10	VGG16	0.9983±0.01	47.4745±1.60
	CIFAR-10	ResNet20	0.9959±0.01	45.4766±2.05
	CIFAR-100	VGG16	0.9964±0.01	45.3336±4.76
	CIFAR-100	ResNet20	0.9977±0.01	45.3974±2.23
	ImageNet	ResNet50	0.9962±0.01	52.1950±4.68
	ImageNet	Inception v3	0.9888±0.01	46.5975±3.08
	ImageNet	DenseNet121	0.9981±0.01	54.5722±2.67

## Data Availability

The data presented in this study are available in ImageNet at https://www.image-net.org/ (accessed on 12 November 20215) and CIFAR-10/100 at https://www.cs.toronto.edu/~kriz/cifar.html (accessed on 12 November 20215).

## Abbreviations

The following abbreviations are used in this manuscript:

ZOO	Zeroth order optimization
FP-ZOO	Fast Patch-based zeroth order optimization
FGSM	Fast gradient sign method
JSMA	Jacobian-based saliency map attack
C&W	Carlini and Wagner
PGD	Projected gradient descent
SimBA	Simple black-box attack (SimBA)
HSJA	Hopskipjump attack
RQ	Research question
